# Electrical Supply Circuit for a Cold Plasma Source at Atmospheric Pressure Based on a Voltage Multiplier

**DOI:** 10.3390/polym13132132

**Published:** 2021-06-29

**Authors:** Ovidiu S. Stoican

**Affiliations:** National Institute for Laser, Plasma and Radiation Physics, 409 Atomistilor Street, P.O. Box MG 36, 077125 Mǎgurele, Romania; ovidiu.stoican@inflpr.ro

**Keywords:** cold plasma, atmospheric pressure plasma, electrical discharges, voltage multiplier, polymers

## Abstract

A cold plasma source operating at atmospheric pressure powered by a voltage multiplier is reported. In addition to its usual high voltage output, there is an intermediate output of lower voltage and higher current capability. A discharge current is drawn from both outputs. The ratio of the current supplied by each output depends on the operating state, namely, before or after the plasma jet formation. The electrical circuit is equivalent to two dc sources connected in parallel, used to initiate and sustain the electrical discharge. The plasma source is aimed to study the effect of cold plasma on the surface of various liquid or solid materials, including polymers.

## 1. Introduction

A wide range of applications is based on the effects of interaction between the cold (non-thermal) plasma generated at atmospheric pressure and the surface of various solid or liquid materials. Developments related to this topic are constantly growing, new research approaches are attempted or are already underway [1,2,3,4,5,6,7]. There are many solutions to obtain plasma at atmospheric pressure, the differences being mainly given by the electrode geometry and type of electrical supply, namely: dc, low-frequency, rf, microwave or a combination of them. A summary of the output parameters, operating conditions and reported applications for various classes of plasma sources is presented in [1]. Depending on the particular design, the magnitude of the power transferred to the electrical discharge and carrier gas nature, the resulting plasma characteristics can be set in a wide range. Because there are no solid or liquid wastes, plasma technologies are environmentally friendly compared with alternative methods based on chemical treatments. Atmospheric pressure operation simplifies the necessary equipment, because no vacuum system is needed.

Cold plasma technology is already an important tool used to process polymers surface for various scientific and technical purposes [6,7,8,9]. By using this technique, the polymer surface characteristics can be modified whereas its bulk properties remain unchanged. The affected layer depth lies in the range 0.005–0.05 μm [8]. There are several areas in which very promising results based on the polymers surface plasma treatment have been reported in the literature, such as: antimicrobial and bioactive coatings [2,10,11,12,13]; surface wettability modification [14,15,16,17,18]; changes of the mechanical properties (roughness, bonding and tensile strength) [15,19,20]; cleaning, improvement dyeing and printing properties [16,21].

This work contributes to the realization of a plasma source of type APPJ (atmospheric pressure plasma jet) aimed to study the effect of cold (non-thermal) plasma on the surface of various liquid or solid materials, including polymers.

Its specific use requires that a plasma source to be operated in a non-transferred arc mode. A plasma jet having well-defined, stable and reproducible parameters under various experimental conditions is also a mandatory demand. Some peculiarities of the experimental conditions may lead to plasma jet interruptions. Generally, to initiate an electric discharge, a much larger electric field is required than the one necessary to sustain it [22,23]. As a result, the quick change of the operating mode for the power supply in order to restart the discharge, followed by a return to the mode corresponding to the discharge sustaining, is necessary. The simplicity of the supply circuit, having in view a possible small-scale plasma source multiplication was also considered. To meet the requirements mentioned above and avoid the difficulties implied by rf or microwave circuits, which need matching networks, a solution based on plasma initiated and sustained by a dc discharge was tested.

## 2. Materials and Methods

### 2.1. Mechanical Layout of the Plasma Source

**Figure 1:**
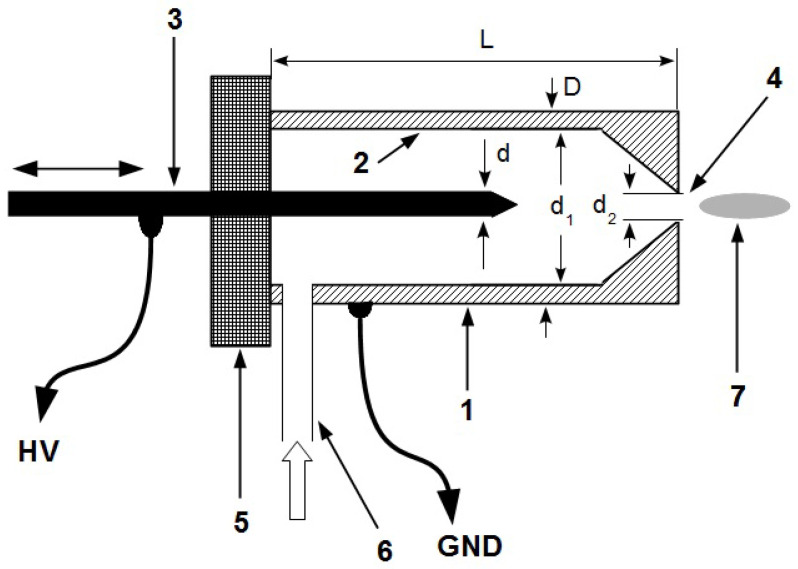
Mechanical layout of the plasma source (not to scale). 1-aluminum block; 2-cylindrical cavity; 3-brass rod; 4-plasma output hole; 5-teflon insulating disk; 6-carrier gas input duct; 7-plasma jet.

The plasma source mechanical layout used for practical testing and operating parameters measurement of the electrical circuit is shown in Figure 1. It consists of a cylindrical cavity terminated with a conical ejection nozzle, drilled along the longitudinal axis of a cylindrical aluminum block. The narrow head of the nozzle is bored and represents the output hole for the plasma jet. The other head of the cylindrical cavity is closed by an insulating disk made of teflon. A sharpened brass rod enters the cavity, along its longitudinal axis, through an orifice existing in the center of the insulating disk. This rod is held in place by the insulating disk and can slide forward or backward, so that its position is adjusted to attain a stable plasma jet of maximum length under the given experimental conditions. For the plasma source, the aluminum block and the brass rod represent the anode (A), which is connected to ground (GND), and cathode (K), respectively. The carrier gas under pressure is introduced into the cavity through a duct that crosses the aluminum block perpendicular to its longitudinal axis. The geometrical parameters of the plasma source, shown in Figure 1, are: d = 3 mm, d${}_{1}$ = 8 mm, d${}_{2}$ = 3 mm, D = 15 mm, L = 22 mm.

### 2.2. Electrical Supply Circuit

**Figure 2:**
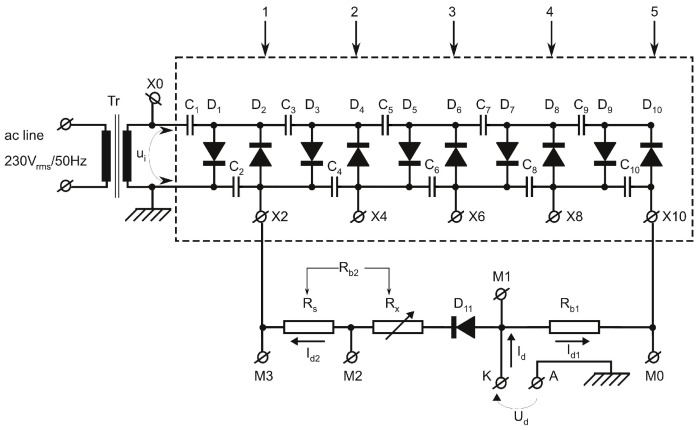
Diagram of the electrical supply circuit. The voltage multiplier, delimited by dashed rectangle, consists of ten identical 1 μF capacitors C${}_{1}$–C${}_{10}$, and ten diodes D${}_{1}$–D${}_{10}$. All multiplier diodes D${}_{1}$–D${}_{10}$ and additional diode D${}_{11}$ are identical, BY6-type. The nominal values of the resistors are $R_{b1}$ = 10 MΩ and $R_{s}$ = 10 kΩ. Resistance $R_{b2}=R_{s}+R_{x}$, where $R_{x}$ may have different values, being used to adjust discharge current. Transformer Tr ensures galvanic isolation between the ac power line and plasma source electrodes. Four voltage dividers are connected to points M0, M1, M2 and M3, to monitor some electrical parameters during plasma source operation.

The electrical supply circuit diagram is shown in Figure 2. Its core consists of a voltage multiplier, delimited by the dashed rectangle. Circuit topology follows the classical one, known as Cockcroft–Walton generator (often called Greinacher multiplier, to give credit to the one who described it first) being a network made up of diodes (D${}_{1}$–D${}_{10}$) and capacitors (C${}_{1}$–C${}_{10}$) which converts the ac voltage applied between point marked X0, conventionally designed here as multiplier voltage input, and ground (GND), to a dc voltage whose magnitude is several times larger than the peak value of that ac voltage. All capacitors C${}_{1}$–C${}_{10}$ are identical, each of them having a capacitance of 1 μF. In addition, all diodes D${}_{1}$–D${}_{10}$ are identical, BY6-type, rated at 6 kV repetitive peak reverse voltage and 1 A maximum forward current [24]. This network can be considered to be formed by a suite of five identical stages connected in series, numbered from 1 to 5, where the first stage is the one at the input, terminals X2, X4, X6, X8 and X10 being the output of each stage. Let the time variation of the ac voltage be applied between point X0 and ground expressed by $u_{i}=\sqrt{2}U_{i}\sin\left(2\pi f_{0}t\right)$, characterized by frequency *f*${}_{0}$ = 50 Hz, peak value $\sqrt{2}U_{i}$ and consequently the root-mean-squared (*rms*) value *U*${}_{i}$. It is preferable to use rms value *U*${}_{i}$, to characterize the magnitude of the ac voltage because common ac voltmeters are calibrated to display this parameter. Based on the operating principle of this type of circuit [25,26,27], theoretically, without any load, taking into account current direction allowed by diodes D${}_{1}$–D${}_{10}$, the dc voltages between terminals X2, X4, X6, X8, X10 and ground are $-2\sqrt{2}U_{i}$, $-4\sqrt{2}U_{i}$, $-6\sqrt{2}U_{i}$, $-8\sqrt{2}U_{i}$ and $-10\sqrt{2}U_{i}$, respectively. Therefore, the maximum negative dc voltage, namely $-10\sqrt{2}U_{i}$, is obtained at terminal X10. Transformer Tr, having its primary winding connected to the ac power line (230 V${}_{rms}$/50 Hz), is used to ensure galvanic isolation between plasma source electrodes and ac power line. Its secondary winding, providing 400 V${}_{rms}$, is connected across point X0 and ground. The cathode K of the plasma source is simultaneously connected to the terminal X10 through the ballast resistor *R*${}_{b1}$ and to the terminal X2 through the equivalent ballast resistor *R*${}_{b2}$ in series with an additional diode D${}_{11}$, respectively. Element *R*${}_{b2}$ consists of two resistors *R*${}_{s}$ and *R*${}_{x}$ connected in series. Resistor *R*${}_{s}$ has a fixed value of 10 kΩ, limiting the maximum discharge current regardless resistor *R*${}_{x}$ value, as well being used for discharge current sensing. Resistors *R*${}_{x}$ can have different values, being used to adjust the discharge current.

### 2.3. Operating Principle

Let the discharge voltage, denoted by $U_{d}$, be the dc voltage applied to cathode K. Note that all dc voltages are referenced to ground and their polarity is negative. Let the discharge current, denoted by $I_{d}$, be the electric current through the plasma source electrodes, from A to K. The current discharge $I_{d}$ is the sum of two components: $I_{d}=I_{d1}+I_{d2}$, where $I_{d1}$ is the current drawn from terminal X10 and $I_{d2}$ is the current drawn from terminal X2, by the electrical discharge. If there is no electrical discharge, then the plasma source electrodes represent electrically an open circuit. Consequently, $I_{d}$ = 0 and the dc voltage applied to cathode is equal to the terminal X10 voltage: $\left|U_{d}\right|\approx 10\sqrt{2}U_{i}\approx$ 5.66 kV, for $U_{i}$ = 400 V${}_{rms}$. To obtain an electrical discharge, implicitly initiate the plasma jet at atmospheric pressure, it is necessary that the X10 terminal voltage to be large enough to generate the breakdown electric field corresponding to the specific mixture air-carrier gas. Therefore, connection to the terminal X10 is only aimed to apply to the electrodes the high voltage necessary to ignite an electrical discharge between them and to initiate or re-initiate, if necessary, the plasma jet. Due to the large value of the ballast resistor *R*${}_{b1}$, the current $I_{d1}$ drawn from the terminal X10 is not sufficient to keep the electrical discharge continuous. It is limited to $I_{d1max}<10\sqrt{2}U_{i}/R_{b1}\approx$ 0.57 mA. The current necessary to sustain the electrical discharge after it is initiated, is drawn from terminal X2. It is limited by the group of resistors denoted *R*${}_{b2}$ ($R_{b2}=R_{s}+R_{x}$), so that $I_{d2max}<2\sqrt{2}U_{i}/R_{s}\approx$ 113 mA. Due to the diode D${}_{11}$ (also BY6-type), the dc current $I_{d2}$ drawn from X2 terminal is null until, in absolute value, the discharge voltage $U_{d}$ becomes lower than the X2 terminal voltage.

Summarizing, in the absence of plasma (no electrical discharge) or in the case of anomalous operation, when $\left|U_{d}\right|>2\sqrt{2}U_{i}$, current drawn from terminal X2, $I_{d2}$ = 0. After the electrical discharge is ignited and carrier gas is flowing, the plasma jet begins to form, the discharge current $I_{d}$ increases whereas the absolute value of the discharge voltage $\left|U_{d}\right|$ decreases until the system reaches a steady state corresponding to the atmospheric plasma glow discharge regime. For the device described here, this state is characterized by a discharge voltage satisfying condition $\left|U_{d}\right|<2\sqrt{2}U_{i}$, at a discharge current $I_{d}$ of the order of ten mA, having as a result a stable plasma jet. To achieve this regime, by varying the resistance $R_{x}$, different values of the ballast resistance $R_{b2}$ were tested experimentally before a suitable range of values was found. The component $I_{d1}$ is not null after the plasma jet is formed and becomes stable, but it can be neglected as compared to the component $I_{d2}$, and practically, $I_{d}\approx I_{d2}$. If, for various reasons, the plasma jet is accidentally interrupted during operation, the diode D${}_{11}$ blocks the current through the terminal X2 and the voltage applied to the electrodes is switched automatically to $-10\sqrt{2}U_{i}$, allowing the re-ignition of the electrical discharge. No supplementary electronic circuit is necessary for that.

## 3. Results

### 3.1. Measurement Setup

**Figure 3:**
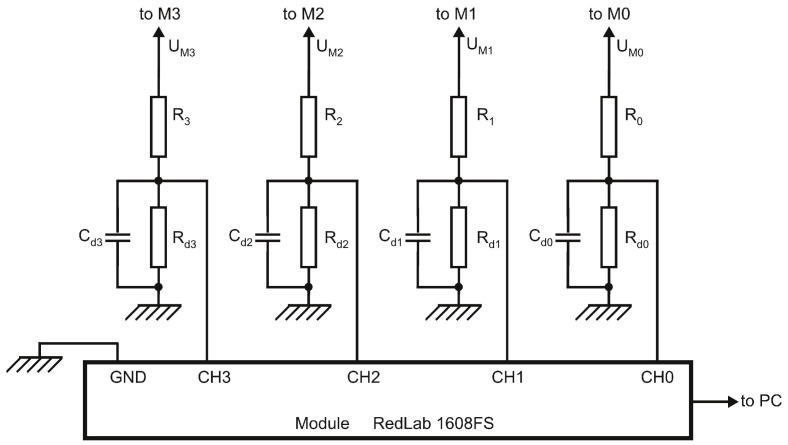
Schematic diagram of the measurement setup. The voltage divider elements nominal values are: $R_{0}$ = $R_{1}$ = $R_{2}$ = $R_{3}$ = 40 MΩ, $R_{d0}$ = $R_{d1}$ = $R_{d2}$ = $R_{d3}$ = 11 kΩ, $C_{d0}$ = $C_{d1}$ = $C_{d2}$ = $C_{d3}$ = 20 μF.

For testing purposes, in order to record voltages values during the plasma source operation, under various experimental conditions, the measurement setup shown in Figure 3 has been used. Four voltage dividers *R*${}_{0}$–*R*${}_{d0}$, *R*${}_{1}$–*R*${}_{d1}$, *R*${}_{2}$–*R*${}_{d2}$ and *R*${}_{3}$–*R*${}_{d3}$ have been connected to points M0, M1, M2 and M3, respectively. To smooth the dividers output voltage ripple, shunt capacitors *C*${}_{d0}$, *C*${}_{d1}$, *C*${}_{d2}$ and *C*${}_{d3}$ have been added. The voltage divider elements nominal values are: $R_{0}$ = $R_{1}$ = $R_{2}$ = $R_{3}$ = 40 MΩ, $R_{d0}$ = $R_{d1}$ = $R_{d2}$ = $R_{d3}$ = 11 kΩ, $C_{d0}$ = $C_{d1}$ = $C_{d2}$ = $C_{d3}$ = 20 μF. A Meilhaus Electronic RedLab 1608FS USB-based Analog and Digital I/O module has been used as a data acquisition system (DAQ). This device performs A/D conversion and transfer data to a personal computer via the USB port. The dividers output voltages are applied to the analog input channels CH0, CH1, CH2 and CH3 of the DAQ system.

Let $U_{M0}$, $U_{M1}$, $U_{M2}$ and $U_{M3}$ be voltages at points M0, M1, M2 and M3, respectively. These are calculated by measuring the output voltage of the dividers *R*${}_{0}$–*R*${}_{d0}$, *R*${}_{1}$–*R*${}_{d1}$, *R*${}_{2}$–*R*${}_{d2}$ and *R*${}_{3}$–*R*${}_{d3}$, and considering the corresponding voltage ratio. All voltage dividers have been previously calibrated in order to calculate the actual voltage ratio. To preclude the effect of ac line voltage fluctuations during measurements, the primary winding of the transformer Tr has been connected to the ac line through an automatic ac voltage regulator. The experimental values against theoretical ones, of dc voltages at terminals X2 and X10, for $U_{i}$ = 400 V${}_{rms}$ (≈566 V peak value), in the absence of plasma, are given in Table 1.

The electrical supply circuit main parameters during plasma source operation result as:

- Discharge voltage representing the dc voltage applied to the cathode $U_{d}$ = $U_{M1}$;
- Voltage multiplier X10 terminal voltage equal to $U_{M0}$;
- Voltage multiplier X2 terminal voltage equal to $U_{M3}$;
- Current drawn from terminal X10 by the electrical discharge calculated as:
  (1) $$I_{d1}\approx \frac{|U_{M0}-U_{M1}|}{R_{b1}}$$
- Current drawn from terminal X2 by the electrical discharge, calculated as:
  (2) $$I_{d2}\approx \frac{|U_{M3}-U_{M2}|}{R_{s}}$$
  by neglecting the current through the voltage divider *R*${}_{2}$–*R*${}_{d2}$ (<0.03 mA).

As can be seen in the next subsection, during normal operation, the discharge voltage $U_{d}$ is about 200 V, so that the current through the voltage divider *R*${}_{1}$–*R*${}_{d1}$ is about 5 μA and may be neglected in comparison with the current drawn by the electrical discharge either from terminal X2 or from terminal X10. Due to the additional load consisting of the voltage divider *R*${}_{0}$–*R*${}_{d0}$, the total current supplied to the outside from terminal X10, denoted $I_{X10}$, is higher than $I_{d1}$. Taking into account that $R_{d0}\ll R_{0}$, it is given by:

(3) $$I_{X10}\approx I_{d1}+\frac{|U_{M0}|}{R_{0}}$$

The second term in Equation (3) is comparable to $I_{d1}$, and the existence of this additional load contributes to the discrepancy between the calculated and measured value of the voltage at terminal X10, shown in Table 1 [27]. However, this leakage current has no significant effect on the plasma source operation and will not be discussed further.

### 3.2. Measurement Results during a Normal Operation Regime

**Figure 4:**
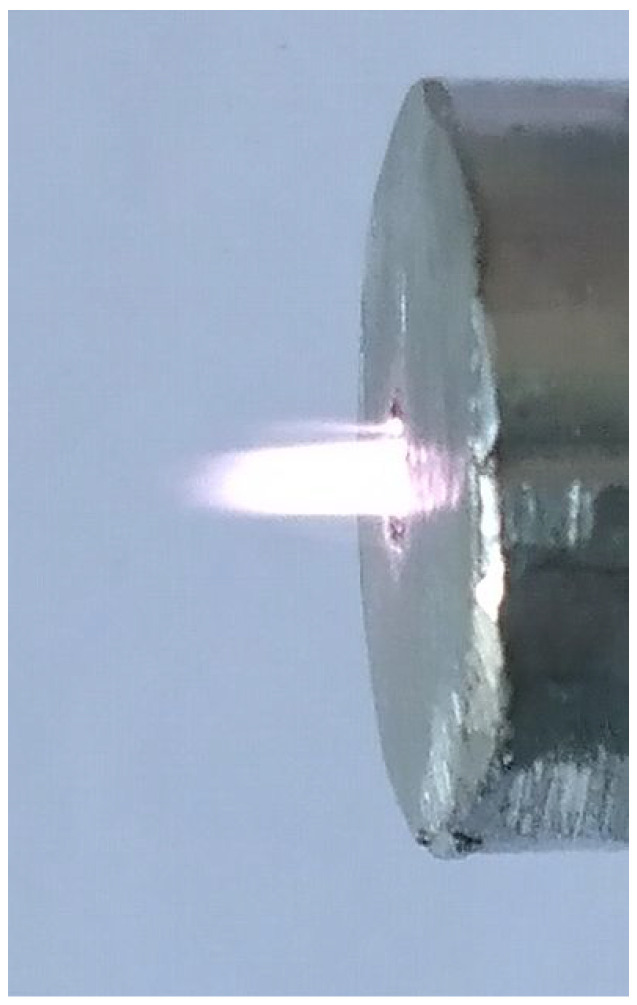
Plasma jet during normal operation. Experimental conditions: $U_{i}$ = 400 V${}_{rms}$, $R_{b2}$ = 20 kΩ, Ar as carrier gas at flow rate of 3 LPM.

The operating regime for various values of the ballast resistance $R_{b2}$ has been tested. Firstly, the electrodes K and A are connected to the supply circuit as shown in Figure 1 and Figure 2. The corresponding electric field is not enough to initiate an electric discharge in the air, at atmospheric pressure. Afterwards, the carrier gas, consisting of Ar, is introduced into the cylindrical cavity of the plasma source by means of a gas flow controller (Alicat Scientific MC-20SLPM). A plasma jet is formed and becomes stable a few seconds after the carrier gas flow is switched on and stabilized at a settled value. By using Ar as carrier gas, at a constant flow rate of 3 LPM, a stable plasma plume of about 5 mm in length was formed. Figure 4 shows a stable plasma jet during normal operation.

The time variation of the voltages $U_{M0}$, $U_{M1}$, $U_{M2}$ and $U_{M3}$ has been recorded for a certain amount of time, by using the measurement setup described above. Data acquisition started after the plasma jet was formed and became stable. Four datasets have been acquired corresponding to $R_{b2}$ = 10, 20, 30 and 40 kΩ. The acquisition time was 300 s, at a scan rate of 1 Hz. In all cases, $U_{i}$ = 400 V${}_{rms}$ whereas carrier gas was Ar at a constant flow rate of 3 LPM. The averaged electrical parameters of the electric supply circuit during the normal operation regime, corresponding to the four datasets, are listed in Table 2. The normal operation regime is considered to be the state in which the plasma jet was formed whereas the voltage and current discharge became relative stable. The average voltage values considered here are calculated as the arithmetic mean of the corresponding data acquired by DAQ:

(4) $${\bar{U}}_{Mi}=\frac{\sum_{k=1}^{N}U_{Mi}\left(t_{k}\right)}{N}$$

where *i* = 0, 1, 2, 3 represents the DAQ channel index, *N* is the total sample number and $U_{Mi}\left(tk\right)$ represents the voltage corresponding to the channel *i* and sample number *k*, acquired at $t_{k}$ seconds from the beginning of the measurement operation, sample number 1 being considered the origin of the time ($t_{1}$ = 0). Therefore, for an acquisition time of 300 s, at a scan rate 1 Hz, *N* = 300 samples and $t_{k}=k-1$ seconds. The averaged currents drawn by electrical discharge ${\bar{I}}_{d1}$ from terminal X10, and ${\bar{I}}_{d2}$ from terminal X2, listed in Table 2, are calculated according to Equations (1) and (2), respectively, where voltages values are replaced by averaged voltages values, calculated according Equation (4).

**Figure 5:**
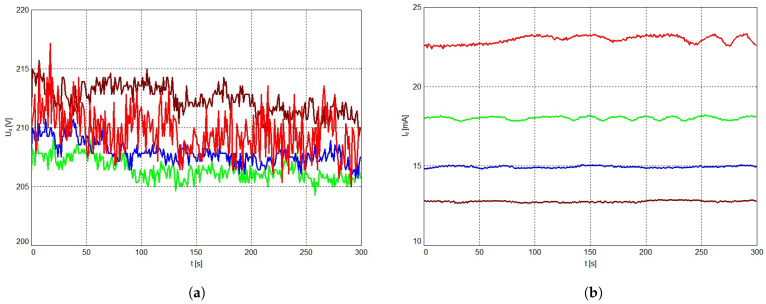
Time variation of the discharge voltage $U_{d}$ (**a**) and discharge current $I_{d}$ (**b**) for ballast resistance $R_{b2}$ equal to 10 kΩ (red), 20 kΩ (green), 30 kΩ (blue) and 40 kΩ (brown).

Figure 5 presents the time variation of the discharge voltage $U_{d}$ (**a**) and discharge current $I_{d}$ (**b**), respectively, for different ballast resistance $R_{b2}$. The graphs shown are based on the datasets used to fill Table 2.

The gas temperature, measured by means of a K-type thermocouple, placed at 2 mm from the output hole of the plasma jet, and the power dissipated by the electrical discharge calculated as: $P_{d}={\bar{U}}_{d}\times {\bar{I}}_{d}$, for different ballast resistance $R_{b2}$, are given in Table 3.

## 4. Discussion and Conclusions

The operation of a cold plasma source powered by a simple electrical circuit based on a voltage multiplier was experimentally demonstrated. Basically, the electrical supply circuit is equivalent to two dc sources connected in parallel, consisting of the two outputs X2 and X10, respectively, of the voltage multiplier. One of them (corresponding to the output X10) provides the very high voltage necessary to initiate the electrical discharge, whereas the other one (corresponding to the output X2) is aimed to sustain the discharge. After a plasma jet was formed, the contribution of the very high voltage source to the discharge current can be neglected as it can be seen in Table 2. A similar approach has already been described in [28]. Unlike the solution described in [28], the circuit presented in this paper has the following distinct features:

- The plasma generation is based on a pure dc electrical discharge. This characteristic combined with a stable regime mode allows to minimize the rf perturbations emission. Technical issues related to the matching circuits required by rf or microwave generators do not exist.
- A very simple electrical supply circuit. The two dc voltage sources switch “naturally”, running simultaneously or separately, as a function of plasma electrical parameters.

No specialized electronic circuit is required for switching between the two dc sources, for example, based on monitoring the discharge current and comparing it with a threshold value.

As shown by the experimental results, the gas temperature can be adjusted in a wide range by modifying the ballast resistor in series with the dc source which sustains electrical discharge, without affecting ignition and stability of the resulting plasma jet. This is an important feature necessary for biological and medical applications [29,30]. In principle, for polymers, the gas temperature must not exceed the maximum service temperature, above which the material subjected to the plasma treatment loses its mechanical properties. For common polymers existing on the market (e.g., Acrylonitrile Butadiene Styrene-ABS, Polyethylene Terephthalate-PET, Polypropylene-PP, etc), this specific temperature is provided by the manufacturer and can be found in various databases usually available online (e.g., [31]). In the case of the experimental samples, containing new materials, biopolymers or living cells, the plasma gas temperature must be maintained as low as possible and, if necessary, progressively increased after a few preliminary tests. As an example, chitosan is one of the polymers intensively studied due to its multiple potential uses [32,33,34,35]. According to [33], during thermal processing, chitosan goes through two or three degradation stages, the first of them occurring at 30–110 °C. For bacterial inactivation, gas temperatures are reported to be about 50 °C [1,6]. Therefore, the gas temperature must be previously observed under various operation conditions, taking into account in particular its variation with carrier gas flow rate and the discharge current, for the given electrodes geometry. Furthermore, the operation conditions will be adapted to set the gas temperature within the acceptable range.

Table 4 presents the maximum relative deviation of the discharge voltage ($\Delta U_{dmax}$) and current ($\Delta I_{dmax}$) from their averaged values for different ballast resistance $R_{b2}$ obtained by picking up the maximum value of the expressions:

(5) $$\Delta U_{d}=\frac{|U_{M1}-{\bar{U}}_{M1}|}{|{\bar{U}}_{M1}|}\times 100$$

and

(6) $$\Delta I_{d}=\frac{|I_{d}-{\bar{I}}_{d}|}{|{\bar{I}}_{d}|}\times 100$$

The results indicate that, after the plasma jet is formed, under the investigated experimental conditions (10 kΩ≤ ballast resistance ≤ 40 kΩ), the discharge voltage and current remain approximately constant, exhibiting small variations around their average values, proving a stable operation regime, whereas the gas temperature can be maintained in an appropriate range. However, for lower ballast resistance, the discharge current and voltage deviation from their average values increases. In a first approach, the dc discharge stability depends on the fulfillment of the Kaufmann criterion [36,37]:

(7) $$R_{b2}+dU_{d}/dI_{d}>0$$

We consider that for lower ballast resistance, the fluctuation in the differential resistance $dU_{d}/dI_{d}$ cannot be fully compensated to satisfy Equation (7), so that the discharge tends to become unstable, as it can be seen in Figure 5.

The plasma jet generated by the device (design shown in Figure 1) has a small section which allows to be applied on a precise material area. On the other hand, in other applications, this could be a disadvantage. As a further development, the possibility to adapt this topology to supply simultaneously multiple plasma jet sources (several, such as the one in Figure 1) will be considered.

Finally, it can be concluded that the electrical circuit described above is a suitable solution for the requirements that were the reason for initiating this work.

## Figures and Tables

**Table 1:** Comparison between calculated and measured magnitude of the dc voltage at terminals X2 and X10 of the circuit shown in Figure 2, having as load only voltage dividers, for the ac voltage applied to the input X0, $U_{i}$ = 400 V${}_{rms}$.

Terminal	X2	X10
Multiple of ac input peak voltage [kV]	−1.13	−5.66
Measured voltage [kV]
Voltage dividers connected to M0 and M4.
Diode D${}_{11}$ and resistor *R*${}_{b1}$ are disconnected.	−1.13	−5.36

**Table 2:** Averaged electrical parameters of the electric supply circuit during the normal operation regime for $U_{i}$ = 400 V${}_{rms}$ and different ballast resistance $R_{b2}$.

Parameter Description	Symbol	Units	$R_{b2}$			
			40 kΩ	30 kΩ	20 kΩ	10 kΩ
Voltage at cathode (discharge voltage)	${\bar{U}}_{d}={\bar{U}}_{M1}$	kV	−0.212	−0.208	−0.206	−0.210
Voltage at terminal X10	${\bar{U}}_{M0}$	kV	−3.01	−2.76	−2.40	−1.84
Current drawn by electrical discharge from terminal X10	${\bar{I}}_{d1}$	mA	0.28	0.26	0.22	0.16
Voltage at terminal X2	${\bar{U}}_{M3}$	kV	−0.71	−0.65	−0.57	−0.44
Current drawn by electrical discharge from terminal X2 (discharge current)	${\bar{I}}_{d}\approx {\bar{I}}_{d2}$	mA	12.77	14.95	18.03	22.97

**Table 3:** Gas temperature $T_{g}$ and power dissipated by the electrical discharge $P_{d}$, for different ballast resistance $R_{b2}$.

$R_{b2}$ [kΩ]	$T_{g}$ [°C]	$P_{d}$ [W]
10	96	4.8
20	78	3.7
30	68	3.1
40	58	2.7

**Table 4:** Maximum relative deviation of the discharge voltage ($\Delta U_{dmax}$) and current ($\Delta I_{dmax}$) from average values for different ballast resistance $R_{b2}$.

Relative Deviation	$R_{b2}$			
	40 kΩ	30 kΩ	20 kΩ	10 kΩ
$\Delta U_{dmax}\left[\%\right]$	1.6	1.4	1.4	3.6
$\Delta I_{dmax}\left[\%\right]$	1.0	0.9	1.3	2.5

## Data Availability

Not applicable.
